# Molecular mechanisms of An-Chuan Granule for the treatment of asthma based on a network pharmacology approach and experimental validation

**DOI:** 10.1042/BSR20204247

**Published:** 2021-03-24

**Authors:** Xiao-Li Chen, Qing-Ling Xiao, Zhong-Hua Pang, Cheng Tang, Qi-Yong Zhu

**Affiliations:** 1 Department of Respiratory Medicine, Affiliated Hospital of Integrated Traditional Chinese and Western Medicine, Nanjing University of Chinese Medicine, Nanjing 210028, China; 2 Department of Respiratory Medicine, Jiangsu Province Academy of Traditional Chinese Medicine, Nanjing 210028, China; 3 Laboratory of Cellular and Molecular Biology, Jiangsu Province Academy of Traditional Chinese Medicine, Nanjing 210028, China

**Keywords:** An-Chuan Granule, Asthma, Inflammation, Network pharmacology, Th17/Treg balance

## Abstract

An-Chuan Granule (ACG), a traditional Chinese medicine (TCM) formula, is an effective treatment for asthma but its pharmacological mechanism remains poorly understood. In the present study, network pharmacology was applied to explore the potential mechanism of ACG in the treatment of asthma. The tumor necrosis factor (TNF), Toll-like receptor (TLR), and Th17 cell differentiation-related, nucleotide-binding oligomerization domain (NOD)-like receptor, and NF-kappaB pathways were identified as the most significant signaling pathways involved in the therapeutic effect of ACG on asthma. A mouse asthma model was established using ovalbumin (OVA) to verify the effect of ACG and the underlying mechanism. The results showed that ACG treatment not only attenuated the clinical symptoms, but also reduced inflammatory cell infiltration, mucus secretion and MUC5AC production in lung tissue of asthmatic mice. In addition, ACG treatment notably decreased the inflammatory cell numbers in bronchoalveolar lavage fluid (BALF) and the levels of pro-inflammatory cytokines (including IL-6, IL-17, IL-23, TNF-alpha, IL-1beta and TGF-beta) in lung tissue of asthmatic mice. In addition, ACG treatment remarkably down-regulated the expression of TLR4, p-P65, NLRP3, Caspase-1 and adenosquamous carcinoma (ASC) in lung tissue. Further, ACG treatment decreased the expression of receptor-related orphan receptor (RORγt) in lung tissue but increased that of Forkhead box (Foxp3). In conclusion, the above results demonstrate that ACG alleviates the severity of asthma in a ´multi-compound and multi-target’ manner, which provides a basis for better understanding of the application of ACG in the treatment of asthma.

## Introduction

Asthma, the most common chronic respiratory disorder associated with a global morbidity rate, affects peoples of all age groups, with particular impact in children [1,2]. Increased smoking and allergy rates, severe air pollution and the aging population have all contributed to an annual rise in the incidence of asthma [3–6]. As indicated by epidemiological data from diverse countries, the prevalence of asthma ranges from 1% to 21%, and its incidence has increased by about 30% over the last two decades [7]. According to World Health Organization (WHO) estimates, 334 million people (4.9% of the world’s population) have asthma, 250,000 asthma-related deaths are recorded each year, and asthma is among the 20 leading causes of disability in children globally [8]. Multiple inflammatory cells are involved in asthma, which is characterized by recurrent episodes of coughing, breathlessness, wheezing and chest tightness [9]. The precise cause of asthma remains unclear to date, but the involvement of gene–environment interactions in its pathogenesis has been widely accepted [10,11]. For decades, the currently accessible therapeutics, including corticosteroids and biotherapies, have been beneficial in the control of asthma, but they are associated with frequently occurring adverse events [12]. Therefore, further study on the course of asthma and on effective prevention and treatment methods has become a priority [13].

Traditional Chinese medicine (TCM), a comprehensive medicinal system, is a critical part of health maintenance for the Asian population and is increasingly popular among Western countries, due to its reliable therapeutic effects and relatively few adverse reactions [14]. According to the theory of traditional Chinese herbal medical science, TCM is a promising preventive and therapeutic option for complicated disorders, including asthma [15,16].

An-Chuan Granule (ACG), one of the TCM formulae, is composed of seven medicinal herbs (Supplementary Table S1), including Ephedrae Herba (Mahuang, MH), Descurainiae Semen (Tinglizi, TLZ), Mume Fructus (Wumei, WM), Sophorae Flavescentis Radix (Kushen, KS), Morindae Officinalis Radix (Bajitian, BJT), Glycyrrhizae Radix et Rhizoma (Gancao, GC) and Ganoderma (Lingzhi, LZ). ACG was first proposed by Professor Zhu Qiyong in the 1990s and has been used to treat many asthma patients. Previous studies have suggested that ACG has favorable therapeutic effect in clinic [17], and significantly suppresses ovalbumin (OVA)-induced asthma in pre-clinical studies. The anti-asthma mechanism of ACG is associated with reduced airway inflammation and tracheal smooth muscle contraction, decreased eosinophil (EOS) number and serum LTE4 level, and recovery of the cAMP/cGMP balance [18]. However, the scientific basis and underlying pharmacological mechanism of ACG remain largely unknown and require further investigation.

Generally, the therapeutic effects of most TCM herbs are mediated by various targets and pathways in the human body, and it is difficult to precisely determine the complex active ingredients of TCM by traditional approaches alone [19,20]. There is therefore an urgent need to develop novel and appropriate strategies to identify these ingredients. In the present study, a comprehensive approach was used combining network-based computational and algorithm-based methods. We explored the active ingredients of ACG and the underlying mechanism of its effect on asthma via network pharmacology analysis and experimental validation.

## Methods

### Network Pharmacology Analysis

#### Data preparation

Data on all ingredients of each herb in ACG were extracted from the Chinese Academy of Sciences Chemistry Database (CASC, http://www.organchem.csdb.cn/scdb/main/slogin) [21], the Traditional Chinese Medicine System Pharmacology Database (TCMSP, http://lsp.nwu.edu.cn/tcmsp) [22] and relevant literature. Data were imported into the ingredient database.

#### Oral bioavailability and drug-likeness screening

Oral bioavailability (OB) prescreening is defined as the degree of distribution of an oral dose of drug into the bloodstream, and it represents one of the most significant prerequisites of oral drug discovery and clinical application. Drug-likeness, a qualitative concept to evaluate the structural similarity of compounds with clinical therapeutic effect in the DrugBank database, is determined early after drug discovery [23]. Wang et al. [24] reported the detailed calculations of these two parameters. In the present study, OB = 30% and drug-likeness index = 0.18 were used as thresholds for selection of candidate compounds. Several compounds, including Lucidone A, Isoprincepin, Americanin A and Dehydroglyasperin C did not meet the above criteria [25–27] but were included as candidate compounds for further analysis due to their widely approved pharmacological activities.

#### Prediction of putative drug targets for ACG

Target identification is considered as a pivotal step in the process of drug exploitation. In the present study, the systematic drug targeting approach proposed by Wang et al. [28] was used to determine potential target medicinal compositions.

#### Collection of the known asthma-related targets

Existing asthma-related targets were obtained from the following five resources: (1) the MalaCards human disease database (https://www.malacards.org) [29]; (2) the Online Mendelian Inheritance in Man database (OMIM, http://www.omim.org) [30]; (3) the Therapeutic Target Database (TTD, http://bidd.nus.edu.sg/group/cjttd/) [31]; (4) the DrugBank database (https://www.drugbank.ca) [32]; (5) and the Genetic Association Database (GAD, https://geneticassociationdb.nih.gov) [33]. Details of these known therapeutic targets are provided in Supplementary Table S2. A total of 188 known asthma-related targets were selected after redundancy deletion.

#### GO-BP and KEGG enrichment analyses

Targets were searched against the Omicshare tools (http://www.omicshare.com/tools) [34] to better understand their roles. A *P*-value of <0.05 was considered as significant, and hypergeometric examination was applied to identify the enriched Gene Ontology (GO) and Kyoto Encyclopedia of Genes and Genomes (KEGG) terms.

### Preparation of ACG

All crude drugs were purchased from Beijing Tongrentang. The following compounds were extracted with boiling water at 1:6 and 1:4 w/v: 6 g MH, 10 g TLZ, 9 g WM, 12 g LZ, 12 g KS, 10 g BJT and 6 g GC. The two filtrates were then combined with the extract in an electric thermostatic water bath and transferred into a vacuum oven to dry. The dried solid extract was crushed into powders and stored at 4°C.

### Establishment of a mouse model of asthma and animal administration

The male BALB/c mice (weight, 18–20 g; age, 7 weeks) were provided by Beijing Vital River Laboratory Animal Technology Co. Ltd. (Beijing, China). All mice were bred in the Nanjing University of Chinese Medicine and housed in a temperature-, humidity- and light-controlled environment. The mice had free access to food and water and were allowed to acclimatize for 1 week before the experiment. All experimental protocols were approved by the Animal Care and Use Committee of Nanjing University of Chinese Medicine (Nanjing, China) and conducted con-forming to the Guidelines for the Care and Use of Laboratory Animals (AEWC-20200801-124). To establish an asthma model (*n* = 32), a 0.5 ml of mixture consisting of 10 mg of OVA, 2 mg of sodium hydroxide or a 0.5 ml of saline solution were intraperitoneal injected into mice in the model group and administration group, respectively, on days 1, 7 and 14. The normal group was intraperitoneal injected with the same volume of saline. From day 21, mice were sprayed with 1% OVA solution and mice in the normal group were sprayed with saline, once a day for 30 min on 12 consecutive days. After this, all mice (*n* = 32) were divided into four groups, including normal group, model group, dexamethasone (Dex) group (1 mg/kg/d, intraperitoneal injection) and ACG group (15 g/kg/d, intragastric administration), with eight mice in each group. On each of the 12 days, 30 min before the atomization, mice in the Dex group were injected with 0.2 ml Dex, those in the administration group were given 0.5 ml ACG solution, and those in the model group and normal groups were administered with 0.5 ml saline. Afterward, animals were killed using CO_2_ suffocation (the flow rate of CO_2_: 30% of the cage volume/min).

### Histological analysis

The left lung was dissected from each mouse in all groups and expanded with 10% buffered formalin. The tissues were then sliced, embedded in paraffin, and 4-μm sections were prepared for morphological evaluation with hematoxylin–eosin (HE) staining and for mucopolysaccharide staining with alcian blue-periodic acid-schiff (AB-PAS). All sections were examined by three individuals in a blind manner. The scoring system for cell infiltration was as follows: 0, no cells; 1, a few cells; 2, a ring of cells 1 cell layer in depth; 3, a ring of cells 2–4 cells in depth; and 4, a ring of cells > 4 cells in depth. In addition, goblet cell hyperplasia in the airway epithelium was quantified based on a five-point system based on the percentage of epithelium occupied by goblet cells: 0, 0% (no goblet cells), 1, <25% of the epithelium, 2, 25–50%, 3, 50–75%, and 4, >75%. The mean scores were calculated from 8 mice [35].

### Immunohistochemistry (IHC)

After deparaffinage of paraffin sections in the 100% xylene and gradient rehydration (100%, 95%, 90%, 85% and 80% ethanol), the endogenous peroxidase activity was blocked by 3% hydrogen peroxide treatment for 10 min, and the sections were washed with phosphate-buffered saline (PBS) twice for 5 min each. The sections were then incubated with a MUC5AC antibody (dilution, 1:500; Abcam) in the primary antibody dilution buffer (Beyotime Biotech, China) for 12 h at 4°C. Following this, the sections were washed with PBS thrice for 3 min each, incubated with the IHC Detection Reagent (HRP, Mouse, Cell Signaling Technology) for 30 min at room temperature, and washed again with PBS thrice for 5 min each. The secondary antibody was detected with the SignalStain R DAB Substrate Kit (Cell Signaling Technology) after which the slides were counterstained with hematoxylin and mounted. To evaluate protein expression, semi-quantitative image analysis was conducted to measure the mean optical density (MOD) using the Image-Pro Plus software version 6.0.

## Assessment of asthma

At the final aerosol inhalation, the mouse behaviors were rated as follows: trembling or nodding (1 point), coughing or deepened breathing (2 points), rhythmic asthmatic cramp (3 points), falling (6 points), and death (10 points). Only the highest score was recorded for each mouse.

### Bronchoalveolar lavage fluid (BALF) cell count

BALF was collected from mice via endotracheal intubation using a catheter by lavage with 0.4 ml of PBS thrice as previously described [36]. After lavage, approximately 1 ml of BALF was recovered. The total cell count in BALF was determined by a hemocytometer according to the trypan blue exclusion method. The eosinophil, neutrophil, lymphocyte, and monocyte counts were then measured in every 300 BALF cells on cytospin smears of BALF samples from individual mice stained with Giemsa stain (Sigma).

### ELISA

The levels of IL-6, IL-17, IL-23, TNF-alpha, IL-1beta and TGF-beta in BALF were detected using the commercially available ELISA kits (eBioscience) in accordance with the manufacturer’s instructions.

### Real-time PCR

The mRNA levels of NLRP3, cysteinyl aspartate specific proteinase (Caspase)-1 and advanced synthesis and catalysis (ASC) were detected using RT-PCR. Briefly, the total RNA of lung tissue was isolated using the Trizol reagent (Invitrogen) according to the manufacturer’s protocols under RNase-free conditions. After this procedure, 1 μg total RNA was reversely transcribed into cDNA using the Hiscript® II QRTSuperMix (Vazyme, China) with the gDNA Eraser following the manufacturer's instructions. The specific transcripts were quantified by quantitative RT-PCR using SYBR Green Master Kit (Bio-Rad) and analyzed with the ABI 7500 RT-PCR system (Applied Biosystems). Gene-specific primers were synthesized by Sangon Biotech and the following primer sequences were utilized:

GGAGGAAGAGGAGGAGGAAA (forward) and ACTGGAAGTGAGGTGGCTGT (reverse) for NLRP3;

GGCTGCTGGATGCTCTGTA (forward) and AGGCTGGTGTGAAACTGAAGA (reverse) for ASC;

CAGACAAGGGTGCTGAACAA (forward) and TCGGAATAACGGAGTCAATCA (reverse) for Caspase-1;

AGGCCGGTGCTGAGTATGTC (forward) and TGCCTGCTTCACCACCTTCT (reverse) for glyceraldehyde-3-phosphate dehydrogenase (GAPDH).

GAPDH was used as the reference to normalize the mRNA levels. The PCR conditions were as follows: at 95°C for 30 s, then at 95°C for 5 s and at 60°C for 30 s, repeated over 40 cycles. The relative mRNA expression was calculated by the comparative *C*_T_ method.

### Western blotting

The levels of NLRP3, ASC, caspase-1, TLR4, P65, p-P65, RORγt, Foxp3 and GAPDH in lung tissue were detected by Western blotting. In brief, tissue homogenates were prepared in the lysis buffer (Beyotime) containing 1 nM phenylmethanesulfonyl fluoride (PMSF) (Beyotime). Subsequently, proteins were denatured, then equivalent amounts of proteins were electrophoresed in the 10% bis-Tris/polyacrylamide gels (Beyotime) and transferred onto the polyvinylidene fluoride (PVDF) membranes (Amresco), which were covered with a 0.45 mm pore-size Millipore filter. The membranes were blocked using blocking solution (tris buffered saline with tween (TBST)) supplemented with 5% skim milk powder and 0.1% Tween 20) for 2 h. The membranes were then incubated with anti-NLRP3, anti-ASC, anti-TLR4, anti-P65, anti-p-P65, anti- receptor-related orphan receptor (RORγt), anti-Forkhead box (Foxp3), anti-caspase-1 and anti-GAPDH antibodies (dilutions, 1:1000; Abcam) in TBST containing 5% bovine serum albumin at 4°C overnight. Following this, the membranes were incubated with horseradish peroxidase (HRP)-conjugated secondary antibody (dilution, 1:10,000) in TBST containing 5% skim milk powder at room temperature for 2 h. Immunoreactivity was then detected using the enhanced chemiluminescence (Applygen Technologies, China). In addition, blots were scanned and analyzed to measure the band intensity using UN-SCAN-IT software version 5.1. Typically, the band intensity was calculated using the following formula: band intensity = sum of all pixel values in the segment selected - background pixel value in that segment.

### Statistical analysis

Data from three independent experiments were expressed as mean ± SD and analyzed using SPSS software version 17.0 (SPSS Inc). One-way analysis of variance (ANOVA) and Dunnett’s post hoc test were used to compare data between all groups and to account for multiple comparisons. A *P* value <0.05 was considered statistically significant.

## Results

### Screening of candidate compounds for ACG

A total of 176 candidate compounds with appropriate OB and drug likeness were collected from the herbal constituents of ACG. In addition, 30 compounds with lower OB or drug-likeness values that exhibited extensive pharmacological activities were also considered as typical components of herbal therapeutics. Thus, these compounds were also selected as candidate active compounds. Finally, 206 compounds from seven herbs were recognized as the ‘candidate compounds’ (Supplementary Table S3). Specifically, the numbers of candidate compounds in MH, TLZ, WM, KS, BJT, GC and LZ were 22, 9, 8, 27, 17, 88 and 55, respectively. Among these candidate compounds, 10 were widely distributed in various ACG herbs with validated diverse biological activities. For instance, quercetin, which existed in four herbs but was absent in KS, BJT and LZ, exerts potent antioxidant, anti-inflammatory and immunoregulatory effects [37,38]. Similarly, luteolin, β-sitosterol, stigmasterol and kaempferol, which were extensively distributed in this formula, also exhibit various pharmacological properties and participate in regulating multiple physiological and pathological processes, such as inflammation, immune response and stress [39–42].

### Prediction of putative targets for ACG

In the present study, the putative targets of candidate compounds were predicted by integrating chemical, genomic and pharmacological information. Altogether 261 putative targets were predicted for the 206 candidate compounds (Supplementary Table S4). The numbers of putative targets related to MH, TLZ, WM, KS, BJT, GC and LZ were 239, 205, 203, 211, 68, 239 and 59, respectively. There were significant overlaps between the seven herbs, regardless of the different numbers of targets associated with each herb, indicating that the different components of ACG might exert congenerous effects by regulating common targets. For instance, both GC and MH are known to affect the occurrence and development of bronchial asthma symptoms through the immune- and inflammation-related pathways (such as the release of inflammatory factors TLR9 and JAK/Stat) [43,44].

**Figure 1:**
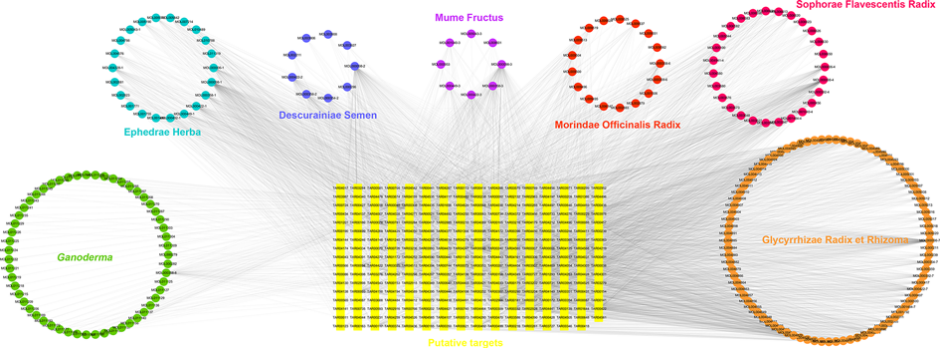
Construction of the ACG compound-putative target network The compound-putative target network was constructed by linking the candidate compounds and their putative targets, the seven herbal constituents of ACG. The nodes representing candidate compounds are shown as polychrome5 polygons and the targets are indicated by yellow squares.

To understand the ACG component-target network from a systematic and holistic perspective, a network map was established using Cytoscape (version: 3.7.2), which comprised 502 nodes and 5170 edges in total (Figure 1). In our network, 56 candidate components were identified to have a median of ≥19 degrees, with confirmed synergistic effects in different herbs and components. Specifically, luteolin, kaempferol and quercetin acted on 68, 74 and 166 targets, respectively, which were subsequently identified as the key bioactive ingredients in ACG. Among them, quercetin has been suggested to alleviate airway inflammation and is beneficial to airway smooth muscle relaxation by modulating key pathways, including the mast cell signaling and calcium ion pathways, which in turn regulate asthma [45,46]. The above findings may explain the pleiotropic effects of TCM herbs.

### Identification and functional analysis of candidate targets for ACG against asthma

**Figure 2:**
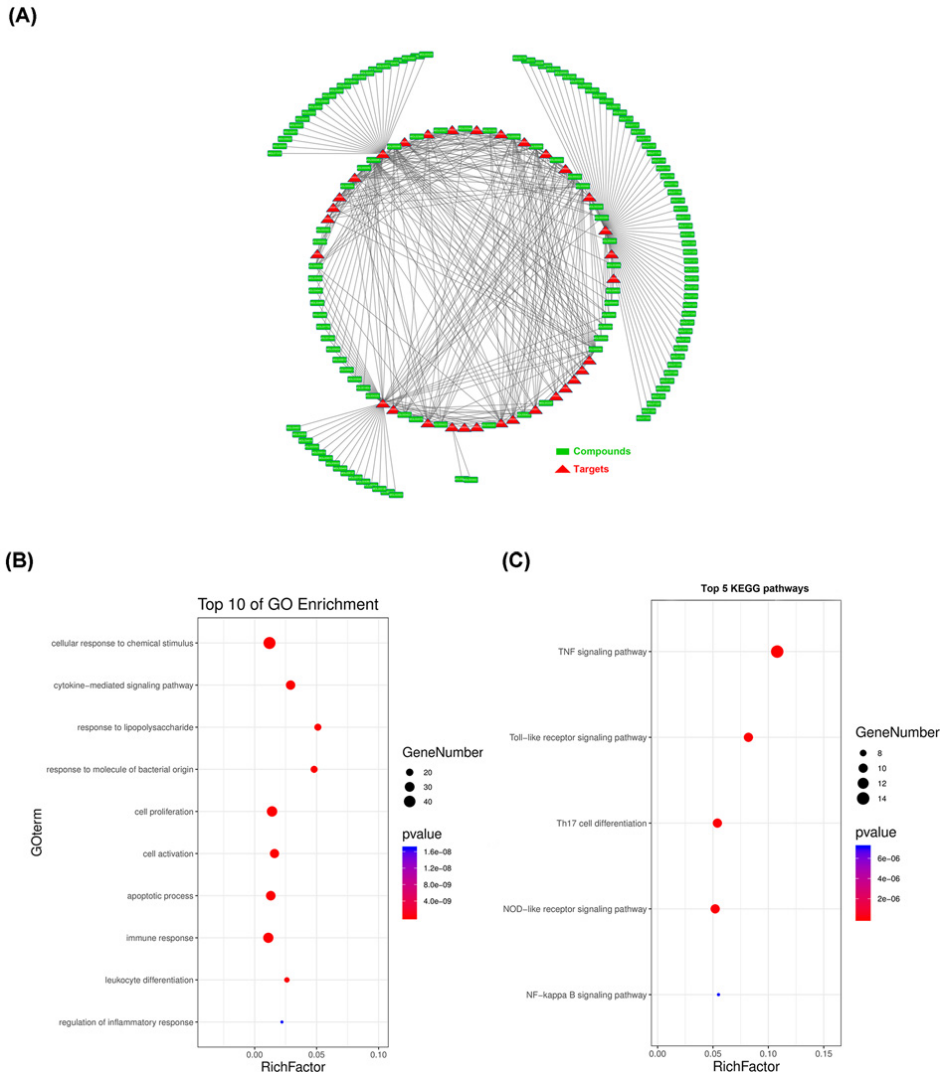
ACG shared 31 putative targets with known pathological course related targets of asthma (**A**) The active ingredients-candidate targets network was constructed by linking the overlapped targets (between ACG putative and known asthma-related) and the homologous candidate compounds of ACG. The nodes representing candidate compounds are shown as green rectangles and the targets are presented as red triangles. (**B** and **C**) 31 candidate targets were searched in Omicshare to gain more insights into their involvement in various GO terms and KEGG pathways. We considered a *P*-value cut-off of **<** 0.05 as significant and applied a hypergeometric test to identify enriched terms.

Our search of five existing resources identified 188 asthma-associated targets. Notably, 31 common putative targets were shared by the seven herbs of ACG, and were the known asthma-related targets identified as candidate targets for the effect of ACG against asthma (Supplementary Table S5). Figure 2A shows the network of active ingredients and candidate targets.

Figure 2B,C show the GO biological processes and signaling pathways related to the 31 identified targets. The biological processes were mainly associated with leukocyte differentiation and proliferation, biosynthesis and release of inflammatory cytokines, cellular response to lipopolysaccharide, regulation of immuno-inflammatory responses and cell death (Figure 2B).

The top five pathways involved included the TNF, TLR, Th17 cell differentiation-related, the nucleotide-binding oligomerization domain (NOD)-like receptor pathway and the NF-kappaB pathway (Figure 2C). These may be key to the pharmacological mechanism of ACG against asthma.

### Effects of ACG on mouse behaviors

**Figure 3:**
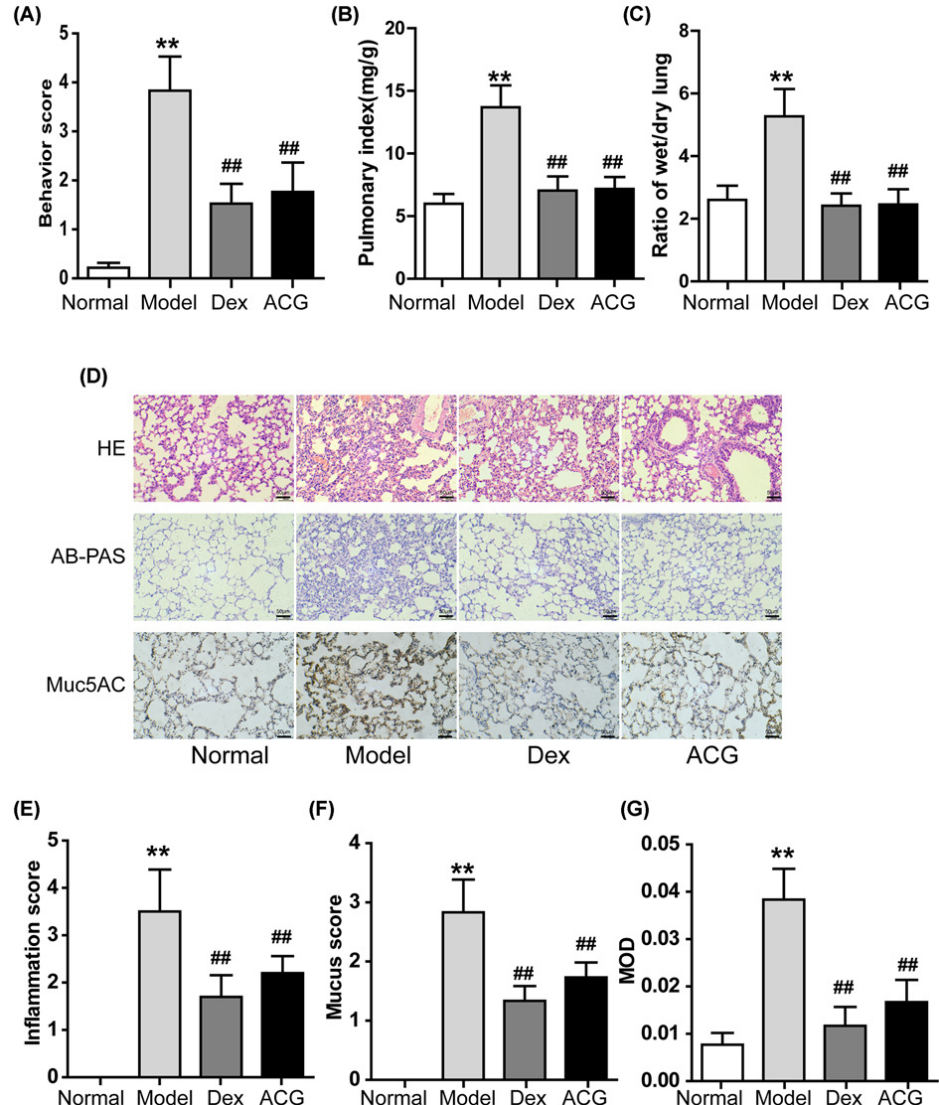
Effects of ACG on the behavioral scores (**A**) lung index (**B**) wet/dry ratio of lung weight (**C**) lung inflammation, mucus secretion and reduced MUC5AC (**D**–**G**) in asthmatic mice. (D) Top: histopathological changes of the lung tissue were observed by HE staining. Middle: the mucus secretion in the bronchus was observed by AB-PAS staining. Bottom: immunohistochemical staining for MUC5AC. (E**–**G) The inflammation score, mucus secretion score and mean optical density (MOD) in each group were shown. All images are shown at 100× magnification. Data are presented as the mean ± SD; *n* = 8 per group. One-way ANOVA and Dunnett’s post hoc test were used to compare data between all groups. ^∗∗^*P*<0.01, compared with the control group; ^##^*P*<0.01, compared with the model group.

During the OVA sensitization period, no significant difference (*P*>0.05) was observed in the mouse behaviors among the different groups. However, during the OVA inhalation process, mice in the model group gradually developed symptoms of trembling, sneezing, scratching, coughing, deep breathing, shortness of breath and even falling down. Additionally, at the final aerosol inhalation, the scores of behavioral changes in ACG and Dex groups were significantly lower than those in model group (Figure 3A).

### Effects of ACG on lung index and wet/dry weight ratio of lung in asthmatic mice

Compared with the control group, lung index and wet/dry weight ratio in the model group were significantly increased (*P*<0.01, Figure 3B,C). Conversely, in the mice given ACG or Dex, lung index and wet/dry weight ratio decreased significantly (*P*<0.05).

### ACG ameliorated pulmonary inflammation, mucus secretion and MUC5AC production in asthmatic mice

As shown in Figure 3D,E, OVA induction marked increased the infiltration of inflammatory cells (mainly eosinophils) into the perivascular connective tissues and peribronchiolar tissues compared with the normal group (*P*<0.05). ACG significantly attenuated the eosinophil-rich infiltration (*P*<0.05). Figure 3D,F illustrate the effect of ACG on mucus secretion. Staining shows marked goblet cell hyperplasia and mucus hypersecretion in the bronchi of mouse lungs from the model group, but not in those of the normal group. In addition, ACG treatment markedly attenuated the OVA-induced mucus secretion. MUC5AC expression was significantly higher in the stained sections of the model group than in the normal, Dex and ACG groups (Figure 3D,G).

### ACG decreased the numbers of inflammatory cells in BALF from OVA-sensitized asthmatic mice

**Figure 4:**
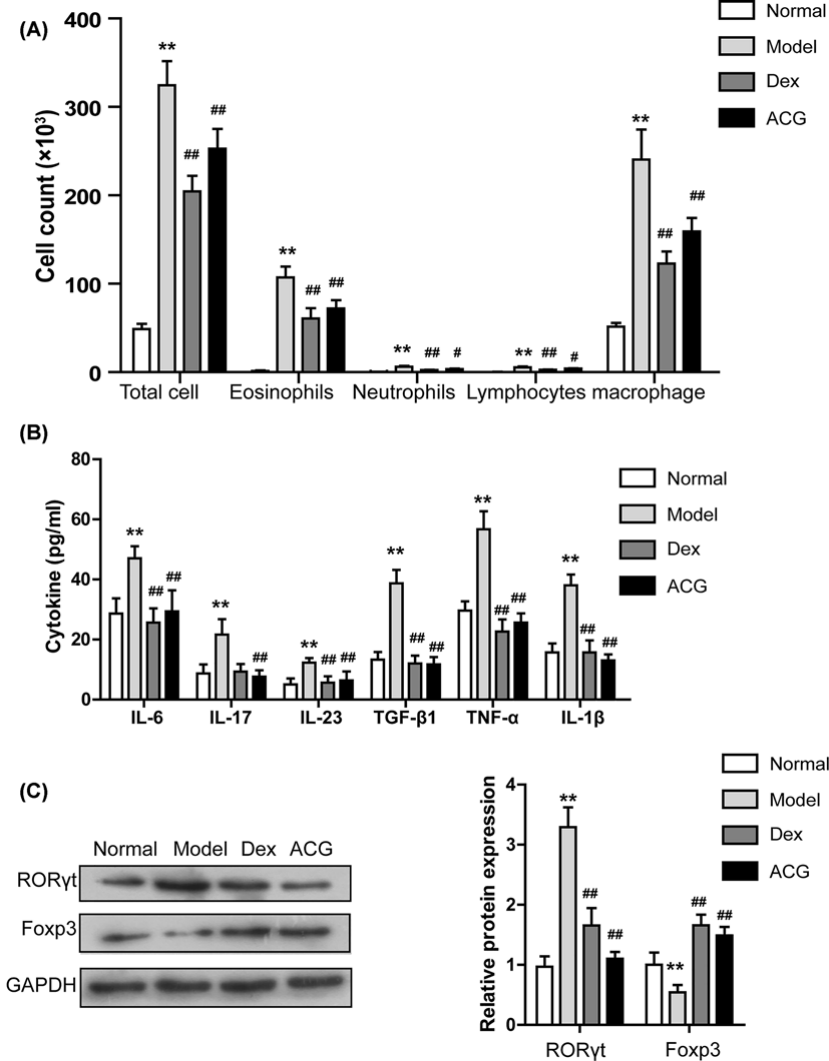
Effects of ACG on the numbers of inflammatory cells (**A**) and pro-inflammatory cytokines (**B**) in BALF, and Th17 cell differentiation-related pathway (**C**) in lung tissue of asthmatic mice. (**C**) Left: immunoblot assay of RORγt and Foxp3 in lung tissues. Right: quantitative analysis of panel. Data are presented as the mean ± SD. *n* = 8 per group. One-way ANOVA and Dunnett’s post hoc test were used to compare data between all groups. ^∗∗^*P*<0.01, compared with the control group; ^#^*P*<0.05 and ^##^*P*<0.01, compared with the model group.

Inflammatory cells were significantly more numerous in mice in the model group than in the normal group. In contrast, the numbers of inflammatory cells in BALF of ACG- and Dex-treated mice were significantly lower than normal (Figure 4A). Analysis of the numbers of different inflammatory cells (eosinophils, neutrophils, macrophages and lymphocytes) in BALF showed a very high number of eosinophils in the model group. The ACG-treated asthmatic mice, however, had significantly decreased inflammatory cells than the model group, with eosinophils particularly reduced.

### ACG reduced the levels of pro-inflammatory cytokines in BALF from OVA-sensitized asthmatic mice

Analysis using ELISA showed that the serum levels of the six cytokines IL-6, IL-17, IL-23, TGF-beta, TNF-alpha and IL-1beta in the OVA sensitized mice were significantly higher in the model group than in controls. However, these cytokine levels were attenuated by ACG treatment (Figure 4B).

### ACG blocked the Th17 cell differentiation-related pathway in OVA-sensitized asthmatic mice

The Th17/Treg balance plays a vital role in the initiation, progression and persistence of allergic diseases like asthma [47,48]. The Foxp3 and retinoic acid RORγt are the key negative and positive regulators of Th17 cell differentiation, respectively [49,50]. The protein level of RORγt was significantly higher while that of Foxp3 was significantly lower in the lung tissue of the model group than that of control group (*P*<0.05, Figure 4C,D). In contrast, RORγt level was significantly lower and Foxp3 significantly higher in lung tissue of ACG- and Dex-induced asthmatic mice than in controls (*P*<0.05, Figure 4C,D).

### ACG inhibited the TLR4/NF-kappaB pathway in OVA-sensitized asthmatic mice

**Figure 5:**
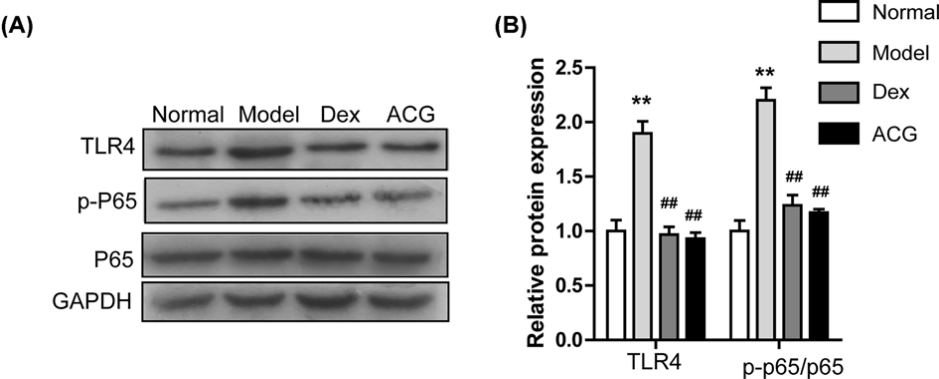
Effects of ACG on TLR4/NF-kappaB pathway in lung tissue of asthmatic mice (**A**) Immunoblot assay of TLR4, P65 and p-P65 in lung tissues. (**B**) Quantitative analysis of panel. Data are presented as the mean ± SD. *n* = 8 per group. One-way ANOVA and Dunnett's post hoc test were used to compare data between all groups. ^∗∗^*P*<0.01, compared with the control group; ^##^*P*<0.01, compared with the model group.

Figure 5A,B show that protein levels of TLR4 and p-P65 were up-regulated in the model group compared with controls. As expected, the administration of ACG or Dex remarkably inhibited the protein expression of TLR4 and suppressed the phosphorylation of P65.

### ACG restrained the expression of NLRP3 inflammasomes in OVA-sensitized asthmatic mice

**Figure 6:**
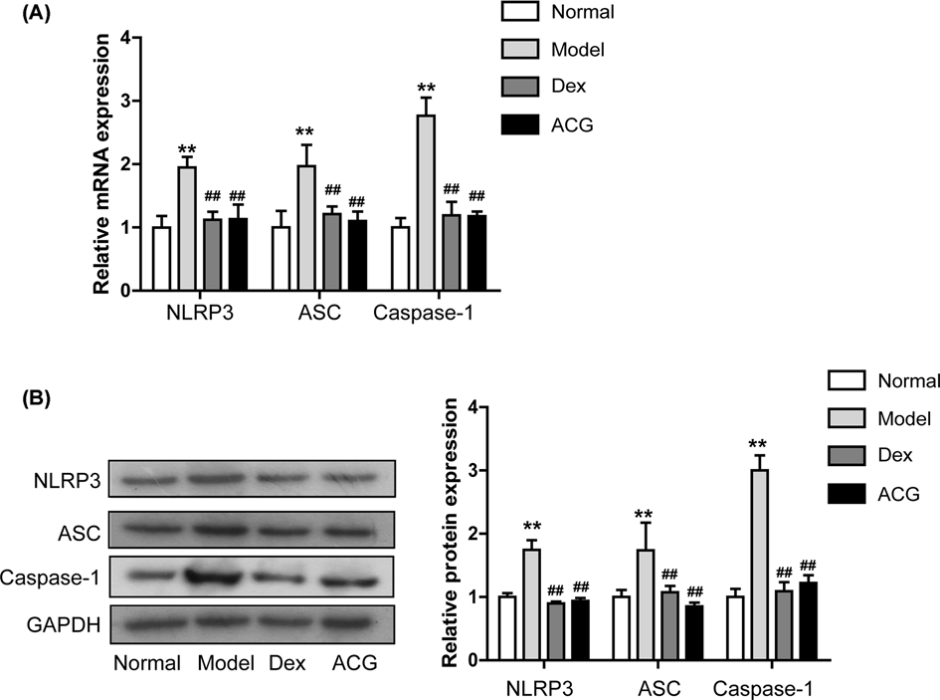
Effects of ACG on the expression of NLRP3 inflammasome in lung tissue of asthmatic mice (**A**) The mRNA levels of NLRP3, ASC and Caspase-1 in the lung tissue were detected by RT-PCR. (**B**) Left: immunoblot assay of NLRP3, ASC and Caspase-1 in lung tissues. Right: quantitative analysis of panel. Data are presented as the mean ± SD. n = 8 per group. One-way ANOVA and Dunnett's post hoc test were used to compare data between all groups. ^∗∗^*P*<0.01, compared with the control group; ^##^*P*<0.01, compared with the model group.

RT-PCR showed that the mRNA levels of NLRP3, ASC and Caspase-1 in lung tissue were significantly down-regulated after ACG treatment compared with those in the model group (Figure 6A). Similarly, the protein levels of NLRP3, ASC and Caspase-1 in lung tissue of ACG-treated OVA-sensitized asthmatic mice were also significantly reduced relative to those in the model group (Figure 6B).

## Discussion

Asthma is a common polygenic predisposing disease of the respiratory system, and is associated with high morbidity and mortality rates [51]. While both TCM and existing pharmaceutical drugs can control the relevant symptoms of asthma [52], TCM herbs may also resolve the underlying cause. The effectiveness of ACG as a treatment for asthma has been confirmed during 20 years of clinical application, but understanding of its pharmacological mechanisms remains unclear. Consistent with clinical findings, our study demonstrated the therapeutic effect of ACG on OVA-induced asthma, improving symptoms such as nose touching, ear scratching, irritability, sneezing, rapid breathing and incontinence in asthmatic mice. ACG treatment significantly decreased lung index and wet/dry weight ratio, inhibited inflammatory cell infiltration, mucus secretion and MUC5AC expression in the lungs of asthmatic mice. In addition, it downregulated the numbers of inflammatory cells and the levels of pro-inflammatory cytokines in BALF from asthmatic mice.

In this study, network pharmacology was utilized to investigate the mechanism of ACG against asthma. Our analysis indicated multiple pathways closely related to the effect of ACG on asthma, including the TNF pathway, the TLR pathway, the Th17 cell differentiation-related pathway, the NOD-like receptor pathway and the NF-kappaB pathway.

Notably, the TNF, TLR and NF-kappaB signaling pathways are classically associated with inflammation, and play critical roles in the pathogenesis of asthma. These pathways promote the production and release of various pro-inflammatory mediators, together with the recruitment of inflammatory-related cells [53–56]. In addition, the TLR4-NF-kappaB pathway has been demonstrated to accelerate the pathological process of asthma by inducing autophagy and apoptosis of tracheal epithelial cells [57]. In this study, ACG significantly inhibited the level of TNF-alpha in BALF and the activity of the TLR4/NF-kappaB signaling pathway.

It is well known that T lymphocytes along with cytokines generated by T-cells are crucial for the progression of allergic asthma, as indicated by multiple studies [58]. Due to the advance in the airway inflammation hypothesis, the imbalance of Th17/Treg ratio has become a paradigm in asthma pathogenesis, and can affect a series of inflammatory cytokines, such as IL-6, IL-23, IL-17 and TGF-β [59,60]. The Foxp3 transcription factor is the key driver for the differentiation and immunosuppressive function of Treg cells [61]. The Foxp3+ Treg cells play essential roles in maintaining the immune homeostasis and in regulating the effector T cell responses. Foxp3+ Treg cells co-express RORγt (in mice) and secrete a higher level of IL-17 ex vivo [62–64]. When there is no second signal from the pro-inflammatory cytokines, Foxp3 can inhibit the function of RORγt and drive the differentiation of Treg cells [65]. When the cells receive a signal from a pro-inflammatory cytokine (like IL-6), the Foxp3 function is inhibited and the Th17 differentiation pathway is induced [66]. As revealed by our results, the levels of pro-inflammatory cytokines IL-6, IL-17, IL-23 and TGF-β increased in the asthma model. In addition, the expression of RORγt exponentially increased but that of Foxp3 significantly reduced. These data indicated an increase in Th17 cells and a decrease in Treg cells, which suggested an imbalance of Treg/Th17, consistent with previous studies. The present study also showed that these pathological changes were reversed by ACG.

Studies on the NOD-like receptor signaling pathways reveal that NLRP3, composed of NLRP3, ASC and Caspase-1, may be an important immune receptor responsible for the auto-inflammatory response [67]. When it is excessively activated, the NLRP3 inflammasomes can lead to serious inflammatory conditions [68,69]. Moreover, previous clinical and experimental studies have demonstrated that the expression of NLRP3 inflammasome is up-regulated in asthma patients and in the OVA-induced asthma model, illustrating the vital role of NLRP3 inflammasome in the pathology of asthma [70,71]. Our results showed that the mRNA and protein levels of NLRP3, ASC and Caspase-1 in the lungs of OVA-induced asthmatic mice were notably down-regulated after ACG treatment, suggesting that ACG might inhibit the inflammatory response in OVA-induced asthma by suppressing the NLRP3 inflammasome. In addition, previous evidence indicates that the activation of NLRP3 inflammasome may lead to increased release of IL-1b [72] and that this increase may be a key factor inducing inflammation and resulting in asthma and may serve as a marker for asthma [73]. In this study, the level of IL-1b in BALF from asthmatic mice was also significantly inhibited by ACG.

## Conclusions

Using network pharmacology and experimental validation, this study highlights the “multi-compound, multi-target, multi-pathway” therapeutic effect of ACG in asthma. Our findings may provide valuable evidence for the further clinical application of ACG as a therapeutic strategy for asthma.

## Supplementary Material

Supplementary Tables S1-S5

## Data Availability

The datasets used and/or analyzed during the current study are available from the corresponding author on reasonable request.

## Abbreviations

AB-PAS: alcian blue-periodic acid-schiff
ACG: An-Chuan Granule
ASC: advanced synthesis and catalysis
ASM: airway smooth muscle
BALF: bronchoalveolar lavage fluid
Caspase-1: cysteinyl aspartate specific proteinase-1
Dex: dexamethasone
EOS: eosnophils
Foxp3: Forkhead box P3
GAPDH: glyceraldehyde-3-phosphate dehydrogenase
GO: gene ontology
HE: hematoxylin-eosin
HRP: horseradish peroxidase
IL: interlukin
KEGG: Kyoto Encyclopedia of Genes and Genomes
MOD: mean optical density
MUC5AC: mucin 5, Subtypes A And C
NF-kappaB: nuclear factor kappa-B
NLRP3: NLR Family Pyrin Domain Containing 3
NOD: nucleotide-binding oligomerization domain
OB: oral bioavailability
OVA: ovalbumin
PCR: polymerase chain reaction
PMSF: phenylmethanesulfonyl fluoride
PVDF: polyvinylidene fluoride
RORγt: retinoic acid receptor-related orphan receptor γt
TCM: traditional chinese medicine
TCMSP: Traditional Chinese Medicine System Pharmacology Database
TGFβ: transforming growth factor-β
TLR4: toll-like receptor 4
TLR9: toll-like receptor 9
TNF: tumor necrosis factor
WHO: World Health Organization
